# Supplementary material for: Complete mitochondrial genomes of four species of praying mantises (Dictyoptera, Mantidae) with ribosomal second structure, evolutionary and phylogenetic analyses
Source: PLoS One. 2021 Nov 4;16(11):e0254914. doi: 10.1371/journal.pone.0254914 (PMC8568281; doi:10.1371/journal.pone.0254914)

**Figure S1. Live habitus images of *Deroplatys truncate*, *Amorphoscelis chinensis,* *Macromantis* sp. and *Deroplatys lobata*.** (A) *Deroplatys truncate*, (B) *Amorphoscelis chinensis*, (C) *Macromantis* sp., and (D) *Deroplatys lobata*.


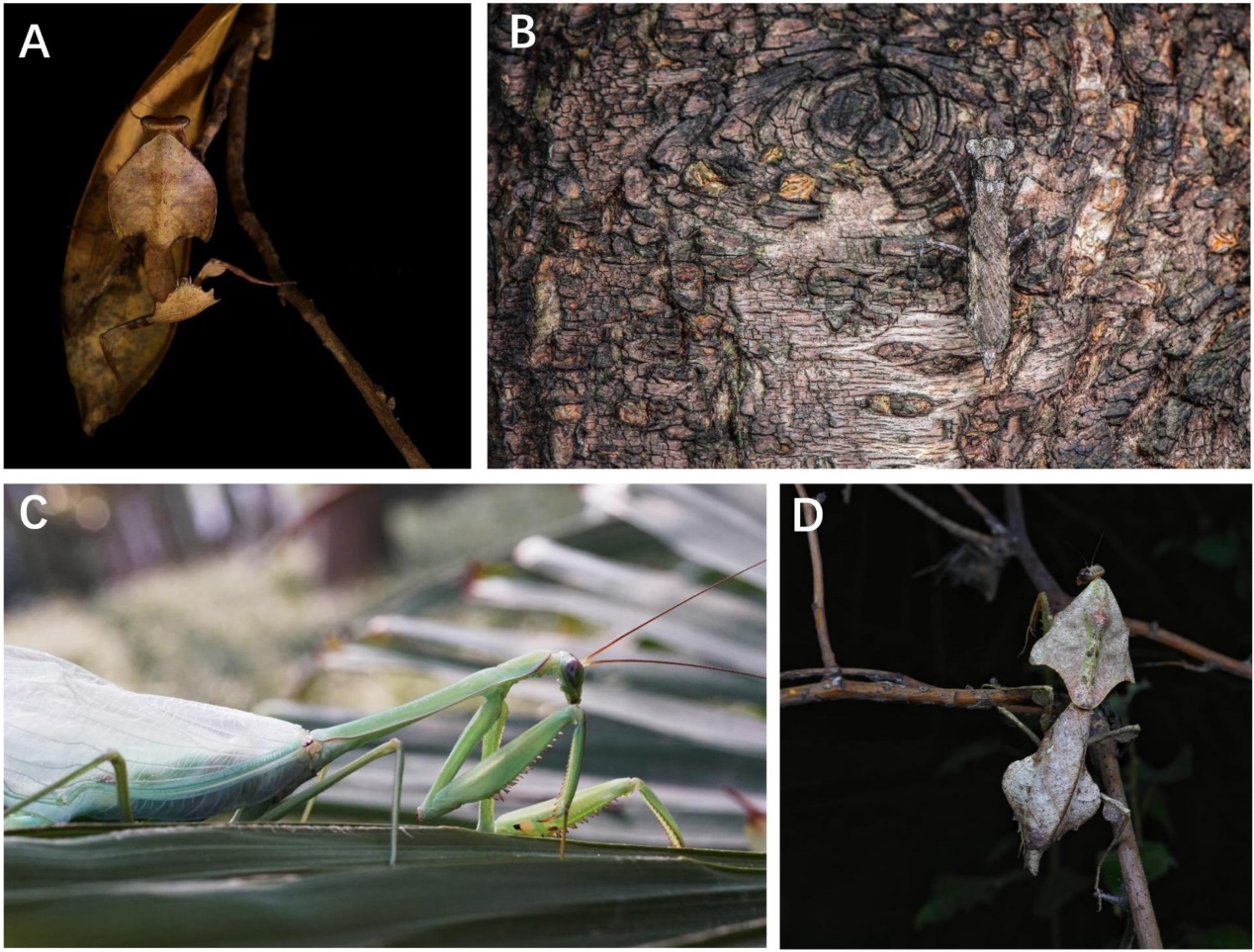

Supplement: S1 Fig — (A) Deroplatys truncate (B) Amorphoscelis chinensis; (C) Macromantis sp. (D) Deroplatys lobata. (DOCX) [file pone.0254914.s001.docx]
