# Supplementary material for: Complete mitochondrial genomes of four species of praying mantises (Dictyoptera, Mantidae) with ribosomal second structure, evolutionary and phylogenetic analyses
Source: PLoS One. 2021 Nov 4;16(11):e0254914. doi: 10.1371/journal.pone.0254914 (PMC8568281; doi:10.1371/journal.pone.0254914)

**Figure S7. Inferred secondary structure of rrnL of *Macromantis* sp*.*** Inferred Watson-Crick bonds are illustrated by lines, whereas the noncanonical interactions are illustrated by dots.


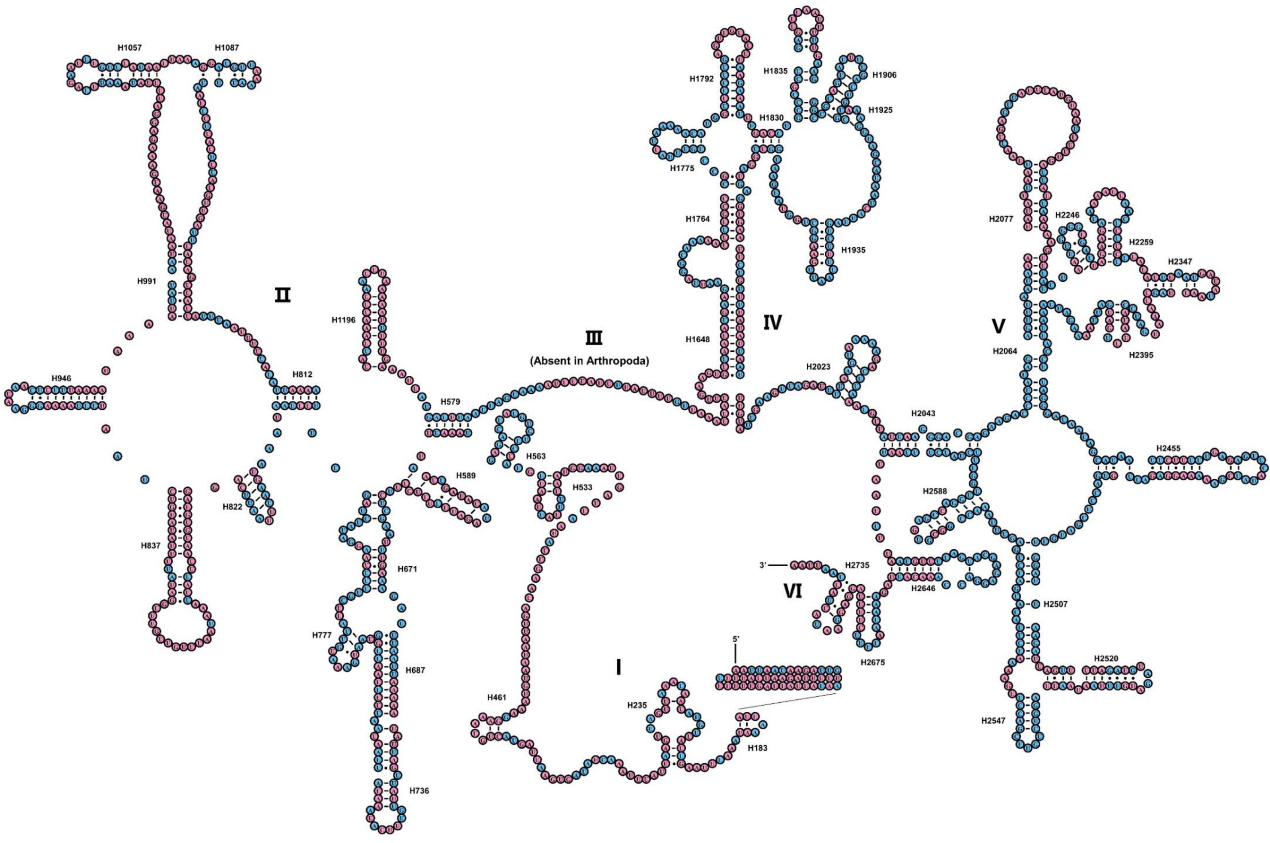

Supplement: S7 Fig — Inferred Watson-Crick bonds are illustrated by lines, whereas the noncanonical interactions are illustrated by dots. (DOCX) [file pone.0254914.s007.docx]
