# Supplementary material for: Complete mitochondrial genomes of four species of praying mantises (Dictyoptera, Mantidae) with ribosomal second structure, evolutionary and phylogenetic analyses
Source: PLoS One. 2021 Nov 4;16(11):e0254914. doi: 10.1371/journal.pone.0254914 (PMC8568281; doi:10.1371/journal.pone.0254914)

**Figure S8. Inferred secondary structures of 18 transfer RNAs (tRNAs) identified in *Deroplatys lobata.*** Bars indicate Watson-Crick base pairings, and dots between G and U pairs mark canonical base pairings in RNA.


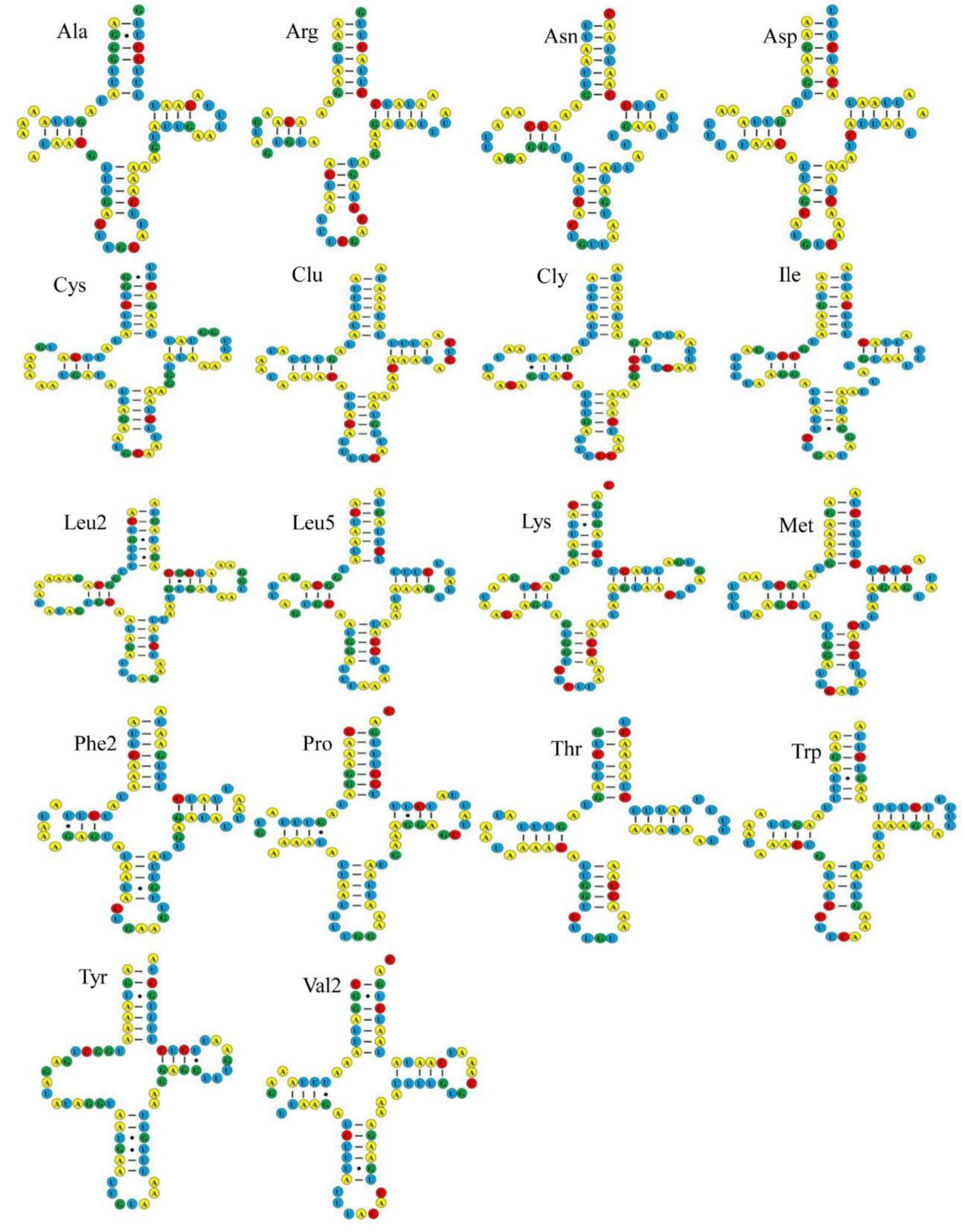

Supplement: S8 Fig — Bars indicate Watson-Crick base pairings, and dots between G and U pairs mark canonical base pairings in RNA. (DOCX) [file pone.0254914.s008.docx]
