# Supplementary material for: Complete mitochondrial genomes of four species of praying mantises (Dictyoptera, Mantidae) with ribosomal second structure, evolutionary and phylogenetic analyses
Source: PLoS One. 2021 Nov 4;16(11):e0254914. doi: 10.1371/journal.pone.0254914 (PMC8568281; doi:10.1371/journal.pone.0254914)
Supplement: S1 Table — (DOCX) [file pone.0254914.s011.docx]

**Table S1.** Verification PCR primers used for amplification of the mitochondrial genome of *Amorphoscelis chinensis, Deroplatys truncate*, *D. lobate* and *Macromantis* sp.

| Primer | Primer sequence (5’-3’) | Tm (ºC) | Amplicon size (bp) |
| --- | --- | --- | --- |
| AC-S1F | ATAATGTTTAATATAATAAGGT | 57 | 3331 |
| AC-S1R | ATTAAATCCTGAAACTAACTCTG |  |  |
| AC-S2F | ATGTTTGATTTATTTCAAGAT | 59 | 3399 |
| AC-S2R | CCCAATAGCCAATCCTATAT |  |  |
| AC-S3F | TATTCTTCTGTAGCTCATATAG | 55 | 3977 |
| AC-S3R | TTCTCTGTACACTATATTCTA |  |  |
| AC-S4F | TCTATACGAAACTAGGTGA | 62 | 5270 |
| AC-S4R | GGTAGGTATACATCTCCATA |  |  |
| DT-S1F | ATCCAATTCTATTTCAACACCT | 62 | 4219 |
| DT-S1R | ATTCATCAACTCTTGTAACAGCCG |  |  |
| DT-S2F | CGGCTGTTACAAGAGTTGATGAAT | 62 | 5217 |
| DT-S2R | TCTTAGGGTCTTCTCGTCTTTTTAT |  |  |
| DT-S3F | ATGGGTGGATGCCTTCTAATCTTTA | 56 | 3798 |
| DT-S3R | ATGTTAGGAACGGTTCAAGAG |  |  |
| DT-S4F | TAGTCTTAGCCCTACCATCAA | 57 | 2190 |
| DT-S4R | AAAATATACACTTCAGGATGACCGA |  |  |
| DL-S1F  DL-S1R | TTTGGTTTCAATTAAACAAGTAAG  ATAAAAAGACGAGAAGACCCTAAGA | 63 4980  57 6998 | |
| DL-S2F  DL-S2R | AGTATCCCAAGAATTAACATAG  AGGTGTTGAAATAGAATTGGAT |  |  |
| MA-S1F | ATAATGTTTAATATAATAAGGT | 57 5721 | |
| MA-S1R | ATTAAATCCTGAAACTAACTCTG |  |  |
| MA-S2F | ATGTTTGATTTATTTCAAGAT | 59 4989 | |
| MA-S2R | CCCAATAGCCAATCCTATAT |  |  |
| MA-S3F  MA-S3R | TATTCTTCTGTAGCTCATATAG  ACCTTATTATATTAAACATTAT | 55 5940 | |
