# Supplementary material for: Complete mitochondrial genomes of four species of praying mantises (Dictyoptera, Mantidae) with ribosomal second structure, evolutionary and phylogenetic analyses
Source: PLoS One. 2021 Nov 4;16(11):e0254914. doi: 10.1371/journal.pone.0254914 (PMC8568281; doi:10.1371/journal.pone.0254914)
Supplement: S2 Table — (DOCX) [file pone.0254914.s012.docx]

**Table S2.** Genes order and ORF features of the complete mitogenomes of *Deroplatys truncate*, *D. lobate*, *A. chinensis* and *Macromantis* sp.

| ORF gene | Strand | *D. truncate, D. lobate, A. chinensis* and *Macromantis* sp. Nucleotide no. | |
| --- | --- | --- | --- |
|  |  | From | to |
| COI | J/N/N/J | 207/ 4654/ 13845/ 1523 | 1682/ 3116/ 12310/ 3061 |
| COII | J/N/N/J | 1845/ 3029/ 12215/ 3116 | 2543/ 2298/ 11493/ 3796 |
| COIII | J/N/N/J | 3512/ 1358/ 10548/ 3857 | 4291/ 573/ 9763/ 4639 |
| CYTB | J/N/N/J | 9206/ 11259/ 4863/ 9377 | 10216/ 10123/ 3727/ 10433 |
| ND1 | N/J/J/J | 11296/ 9103/ 2735/ 10628 | 10430/ 10038/ 3610/ 11465 |
| ND2 | J/N/N/J | 14301/ 5891/ 15078/ 790 | 14894/ 4860/ 14053/ 1207 |
| ND3 | J/N/N/J | 4446/ 430/ 9667/ 4791 | 4727/ 149/ 9338/ 5057 |
| ND4 | N/J/J/J | 8290/ 12178/ 5784/ 7127 | 6959/ 13515/ 7121/ 8443 |
| ND4L | N/J/J/J | 8535/ 11939/ 5536/ 8458 | 8284/ 12184/ 5790/ 8703 |
| ND5 | N/J/J/J | 6870/ 13595/ 7187/ 5380 | 5224/ 15334/ 8929/ 7044 |
| ND6 | J/N/N/J | 8754/ 11696/ 5366/ 8884 | 9203/ 11259/ 4863/ 9426 |
| ATP6 | J/N/N/ | 2835/ 2041/ 11228/5884 | 3512/ 1361/ 10548/6426 |
| ATP8 | -/-/N/- | -/-/11380/- | -/-/11222/- |
