# Supplementary material for: Complete mitochondrial genomes of four species of praying mantises (Dictyoptera, Mantidae) with ribosomal second structure, evolutionary and phylogenetic analyses
Source: PLoS One. 2021 Nov 4;16(11):e0254914. doi: 10.1371/journal.pone.0254914 (PMC8568281; doi:10.1371/journal.pone.0254914)
Supplement: Attachment — Submitted filename: R2-Reply letter-GS2.docx [file pone.0254914.s014.docx]

26/06/2021

Dear Editor,

Thank you very much for your email message regarding our manuscript entitled “Complete mitochondrial genomes of four species of praying mantises (Dictyoptera, Mantidae) with ribosomal second structure, evolutionary and phylogenetic analyses”.

We appreciated the valuable comments from the reviewers and editor to revise and improving our manuscript. Generally, we have incorporated all suggestions and comments into this revision. More specifically, our adjustments are stated below in a point-by-point manner.

We hope that this revised version can be accepted for publication.

Most sincerely,

All authors

Reviewer #2:

Thank you for your thorough responses and edits to your manuscript based on my comments. I think the manuscript has been improved. I do have a few comments that still should be addressed, based on my first round of comments:
1. I still think the paragraphs in the Introduction are a little disconnected. Perhaps switch paragraph #2 (on the utility of mitogenomes for mantid phylogenetics) and paragraph #3 (on mantid taxonomy).

Reply: We agree and have switched the paragraph #2 and paragraph #3. See lines 46-68.

2. I think you should add the AT% to the main text, not just in a supplement.

Reply: Indeed, we have added the AT% information to the main text; see in Table 2.

3. I’m still somewhat skeptical that the atp8 gene is actually missing, but your combination of NGS and PCR results are more convincing. It is difficult for me to fully evaluate this claim without access to the sequence data; but as is stated in the manuscript, atp8 and other genes are missing from other taxa. Is this the first case of missing mt genes in mantises?

Reply: Thanks for your suggestion. Here we attach mitochondrial sequencing results for *D. truncate* species, and we really did not identify the atp8 gene. So, we imply that atp8 gene may have been lost. Yes, from our sequence of *D. truncate* and *D. lobata*, we can confirm the atp8 is lost. This is the first case of missing Mt gene in mantises.

TAATTATCCCATGAAAGATTAGTATTAAACCAATAACTCTAAATTTATTTTAAATAATAGAGTAAAATGCCTGATTTAAAAGGAATATCATGATAGGATAAAATATGTAAATTAATTACTTTTACTAATATTATCATTACACATAACAGAGTTAAACTGTTTCCTAAAATATCAAAAATTTTTGTGCATCCTACACTAAAATATAGAAAAGTAAGCTAAATTTAAGCTAATGGGTTCATACCCCATATATAGAGTTTACACTCTCTTTTCTAGTGCCCAACAATTCGATAAAAATCCTCTTTTTAATAACTTTAATTAGAGGTGTGTTAATTTCATTATGTGCTAATTCATGAATAGGAGCATGAATAGGATTAGAAATTAACTTACTCTCCTTCATTCCTTTACTTTCTTCCAGAAAAAACTTGTTCTCAACTGAAGCTTCACTTAAATATTTTCTTATTCAAGCTATTTCATCATCTACATTTCTATTTCTAATTATGATAAAAATTAATATCCAAGAAATGTTTTATCTAATAAAATTTAATAATTGAAACACCTTAATTACAATTCCTATTCTAATGAAAATTGCATCTGCACCTTTTCATTGATGATTACCTTCCGTGATAGAAGGTTTATCTTGAATAAATTGTTTTATTATTCTGTCAATTCAAAAAGTAGCCCCATTAATATTCATTTCCTACTTAATCACTAATAACTTCTTTATTCAAATCATCATTACAATTTCTGCAATTATAGGAGCTATTGGAGGTCTCAATCAAATTTCTCTACGAAAAATTTTATCATTTTCATCTATCAACCATATTGGATGAATATTAACAACCATAATTATAGGTTCTAACTTTTGAATAACATACTTTATCATTTATACGGTAAATATTATCCCTATTATTTTTATGGTAAACAAAATAAACCTATCTTTTATTCCTCAGACCTTTAATTCCTTCAATAACAAAAAAATTATTAAATTCATTCTATTTATCTCCCTCCTTTCTTTAGGGGGCCTCCCTCCTTTTATTGGTTTTTTCCCAAAATGAATTATCATTCAAATTATAATCCAAAATTTAATAATTCTTACATCAATAACCCTAATTATATCCTCCCTTTTAACCCTTTATTATTATCTACGAATTATTTACACAACATTAATAATCACAAATTCAGAAATAACTTGAATAGCAATCTACTCAAATAACAACTTAGGAAAAAGAACATTTCTTTTCTTATTCATTCTTATTTTTGGTTTATCAATCTGTACACTTATCTTAACAATCTATTAAGATTTTAAGTTAACCAAACTAATATCCTTCAAAGTTATAAATAAAGTGATTCATCTTTAAGTCTTAGTATTACTTACACCTTTAGAATTGCATTCTAATATCATCTCCATGAATATAAAACTTTAGTAAAAGAGATAATAATCTCATTAATAAATTTACAATTTATTACCTAATATCAGCCATTTTACTTTTTTTTTTGCAACGATGATTATTCTCAACAAATCATAAGGATATTGGAACATTATATTTTATTTTTGGTGCATGGGCAAGTATATTAGGAACATCTTTAAGAATTTTAATTCGAACAGAGTTAAGACAACCCGGATCTTTAATTGGAGATGATCAAATTTATAATGTCATTGTAACAGCTCACGCTTTTATTATAATCTTCTTTATAGTTATACCCATTATAATTGGTGGATTTGGTAATTGATTAATTCCTTTAATACTTGGAGCTCCAGATATAGCCTTTCCTCGAATAAATAATATAAGATTTTGATTATTACCTCCTTCTATTCTATTTCTTATTATCGGAAGAGTTGTAGAAAGAGGAGCTGGTACAGGATGAACAGTCTATCCCCCTCTTTCAGCCAGAATTGCTCACGCCGGCCCCGCAATTGATTTAACCATTTTTTCCTTACACTTAGCTGGAATATCAAGAATTATAGGAGCTGTAAATTTTATCACAACTATAATTAACATAAAATCAATTCATATAAATCATACTCAAATTCCTTTATTTGTTTGATCTGTAGGAATTACAGCAATTCTTCTTCTTTTATCTCTCCCTGTACTTGCTGGGGCAATCACTATACTTCTAACTGATCGAAACTTAAATACATCCTTTTTCGACCCTGCTGGAGGGGGGGATCCTATTCTCTATCAACATCTATTTTGATTTTTTGGACATCCTGAAGTATATATTTTAATTTTACCAGGATTTGGTATAATTTCTCATGTCATTTCTCATGAAAGAGGAAAAAAAGAAGCTTTTGGAAATTATGGAATAATTTGAGCTATATCAGCCATTGGTTTTCTGGGGTTTATTGTATGAGCTCATCATATATTTACAGTAGGAATAGATGTAGATACACGAGCCTACTTTACAGGAGCTACTATAATTATTGCTGTTCCTACGGGTATTAAAATTTTTAGTTGACTCACAACTATATACGGAACAAAAATAATTTATAGTGTAGTATCTTTATGAGCATTAGGATTTATTTTTTTATTTACAGTTGGAGGTCTTACAGGAGTAATTTTAGCCAATTCAGCTATTGATATTATTCTTCATGACACTTATTATGTAGTAGCCCATTTTCACTATGTACTTTCAATAGGAGCCGTTTTTGCTATTATAGCAGGGTTCATTCATTGATATCCCTTATTCACTGGATTATCATTAAATCCTAATTGATTAAAAAGTCAATTTTTCACAATATTTGTAGGAGTTAATTTAACATTTTTTCCACAACATTTCTTAGGATTAGCTGGAATACCTCGACGCTATTCAGATTACCCTGACGCCTATAGATCATGAAACTTCTTATCATCTGTAGGAGCAATAATTTCTTTCGCTGCTGTTATCATATTCATTCTTATCTTATGAGAAAGAATTACCTTAAATCGATTTATATTATTTTCATCTCAAATAAATAGATCAATTGAATGAATTCATAATTTTCCCCCAGCTGAACACACCTACAATGAACTAACTCTAATTACAAATTAAAACCTAAATTGTTGATAATTTTCCTCATTTCTAATGTGGCAGAATAGTGCACTGGGTTTAAGCTCCAAAAATAAAGATAAACTTTTTTTAGAAGTTATTTTAATGGCTACAAATGCAAATTTAGGATTTCAAGATAGAGCCTCCCCCTTAATAGAACAACTCATGTATTTTCATGATCATTCCATATTTATTATTACTATAATTGTAATTACAGTAAGTTATATAATTATAGCTTTAATAGTAAATAAATTTTCTGATCGTCATGTTATAGATGGTCAATATTTAGAAATTTTTTGAACAGTTCTTCCAGCCATAGTTCTAGTCTTCATCGCTCTACCTTCTCTACGGATTTTATACCTAATTGATGAAAATACAAATCCAACATTAACCTTAAAAACAATTGGTCATCAATGGTATTGAAGTTACGAATATTCAGATTTTACCAATATTGAATTTAACTCTTACATAATTCCACAAAATGATTTAAATCTATTTAACATGCGTTCACTTGAAGTTGACAATCGAACATCATTACCCATAAATACCTTAACACGAATTTTAATCACCTCAGATGATGTTATTCATTCTTGAACAATTCCGAGAATTGGAGTAAAAGCTGATGCTACTCCGGGACGATTAAATCAAGCAACATTTTGATTTAATCGTCCCGGAGTATTTTATGGTCAATGTTCAGAAATTTGTGGAGCAAATCACAGATTTATACCTATTGTAATTGAAAGAACTTTGATTAATAATTTTCTTAGTTGAATCTTAAATTATATTGAATCACTAGATGACTGAAAATAAGTGATGGTCTCTTAAACCATATCATAGTAACATAATAACTACTTCTAGTGATTGACTAACAATTTATCAAGAAGTTAGTTAAAAAATAACATTAGTATGTCAAACTAAAGTCATTATCATTTAATACATCTTTATACCCCAAATAATACCCCTAAATTGGCTAATCCTATTTTACATTGTTTCTACTAGATTAATTTTTTTTAATGTAATAAATTTTTTTATATTTTCCCACAAAATCCCATTAACTTCAAATAAAATTTTACTAAAAACCCTAATTTGAAAATGATAACCAACCTATTTTCAATTTTTGATCCCTCTTCAAATTTTATAAACCTATCAATAAATTGACTTAGAATTTGAATCGGATTATTATTATTTCCTTCCTCGATATGGTTAATTTCATCACGAAATAAAACCCTTTGAAGTTTTATTTTAAGTAAACTTCATGAAGAATTTAAATTATTAATTGGTAAAAAAAAAATTAACAAAGGATCAACATTCATATTTATTTCAACATTTTTACTTATTATATATAATAATTGTATAGGATTATTTCCATATATTTTTACTGGGACAAGTCATATAGCTATAACTCTATCTTTTGCTTTACCTTTATGACTAAGATTTATACTCTTTGGTTGAATTAATAACTCTAATCACATATTTATTCATTTAGTTCCTCAAGGAACTCCAAATATATTAATACCTCTTATAGTTTGTATTGAAACAATTAGAAATTTAATTCGTCCCGGAACTTTAGCTATTCGACTCGCAGCAAATATAATTGCAGGACATTTATTAATAACCCTCCTAGGAAATTCTGGGAGAAACATTATAGATTCATTTTTACCCCTATTAATTTTAGTTCAAATTATACTTTTAACTCTAGAATCCGCAGTTGCTATTATCCAATCATATGTTTTTGCAGTATTAAGTACTTTATACTCTAGAGAAGTAAATTAATAATGATAATACACACTAATCACCCCTACCATTTAGTTACTTATAGTCCCTGACCTATTATAACTACTTTAAGAATCATAATCATAATATTAGGTTTTATTAAATTTTCTTATGAGTTTAGTGAAAAATTTATGCTATTAGGAACTTTAATTTTAATTTTAATTACTACTCAATGATGACGTGACGTTGTACGAGAAAGTACATATCAAGGATTACACACTAAAAAAGTAATCTTTGGACTACGATGAGGAATAATTTTATTTATTATTTCAGAAATTTTTTTCTTTGTATCTTTTTTTTGAACTTTTTATCATAGAAGCTTAACTCCTACTATTGAATTAGGGTCCTTTTGACCACCTCAAGGAATCTGACCCTTTAATGCTCTTCATGTTCCTCTTCTTAATACAACGGTACTTTTAGCATCAGGCATCACTATTACATGAAGTCACCATGGACTATTAATAAATAATTATAATCAAGCCACCCAAGGATTAATATTTACCATTATCCTTGGGATTTATTTTACCATCTTACAACTCTATGAATATTATGAAGCTCCGTTTACAATTGCGGATTCAGTTTTTGGGTCAATCTTTTTTATAGCAACTGGATTTCATGGACTTCATGTAATTATTGGAACTACATTCTTAGTTACATGCTTATTTCGAATAATTTATAAACATTTTACATCTATTCACCACTTTGGTTTCGAGGCAGCAGCCTGATATTGACATTTTGTGGACGTAGTATGATTATTCCTGTACATTTCTATTTATTGATGAGGGAGATAAATCCAATTTATTTAGTATAAAAGTACAATTGATTTCCAATCAAAAAGTCTATATTTAATTAGAATAAATAATTAAAATTTTAATTTTTATCTCATTCATTACTATATCAATTACCTTAACAATCATATTACTAACAAATTTCTTGTCAAAGAAAAAAATTGAAGACCGAGAAAAAAATTCACCTTTTGAATGTGGATTTGATCCGATTAGATCCTCGCGCCTTCCCTTTTCTTTACGTTTCTTTTTGATTGGAGTAATCTTTTTAATTTTTGATGTAGAAATCGCCTTTATCTTACCAATAATTATCATTCCTCTTACATCAAAAATAACATCTTGAATATCTACTAGAATTATATTCTTATTGATCTTAACAACTGGTTTATTTCATGAATGAAATCAAGGTTCTCTCGACTGAGCAACTTAACTTTATAAGGGTTATAGTTAAAAATAACATTTGACTTGCACTCAAAAAGTATTGAAATATCAATTTTCCTTATTATAAGTAAGAAGCAAATTCATTGTAATCAGTTTCGACCTGATAGTAAGATATTCATATCCTTATTTGTTGATTAATTGAAACCAAATAGAGGTATATCACTGTTAATGGTAAAATTGAAATTAATGCTTTCCAATTAAGAAAATGTGTAGATCGAATATAAGTTGCTAATTTATTATTCAAGTGGTTTAATCCCATTTACATTTTAATTTATATAGTTTAAATAAAACATTACATTTTCATTGTAAAAATAAATTTTTCAATTTTATTAATAGTAAGTACTCTATTTAAAGATAAATTAGTTATCTCAATAACAGCTTCAATGTTATACTCTCTATAAGATATTTAAATAAATACAAAATATTATTATAAGTAAAATACTAAAAAAAATCATTAAATAAGATTTTAAATCATTATATTGAAATCATTGATTAAAACATCTTAATTTTATCAAAATATAATATAAATTTTGTGCTCCAAAATATTCTCTTCAGCCTAAATCAATATATATCATTGAATTTAAACTAATAATTAAAGGTATTTTTCTTACTCCTTTAGTAAAAATTAAAGGTATATATCATATTGAAGCAAAAAAAATTGTCATTTTATAATACTTAAATGTTGAAAAATAATAATTTATTCTATACTTAAATATAATTCTTCCTAATCATAAACCTACAAATCTACCTAATAAAGGTATTATTTTCATTATTAATGTCATATAAATTATATAGGGAGTAAGAAAAATAATCCAATTTAATATCCCACCTCCAATAATTGCAAATAATATTAAACCTAATATTCCATATACTATTATTCAACTTTCATTTAATTTACATATTGATATTATATTAAAATCTCCTCATAATACATAATAGGATAAACGAAAAGAATAACTTACTGTTAAACCTGTTGAAAAAAAAAATAAAACATATATAAAGATATTTAAATTTCTTAAAGATAATATTTCTAAAATTATATCTTTTGAATAAAATCCTGCTAAAAAAGGTATACCACATAAAGCAAAATTTGATACTATAAAACAAGATGAAGTAAAAGGCATAAATATTACTAAATTTCCTATAAAACGAATATCCTGTAAATTTTTTATTCTATGGATTATTATTCCTGTACACATAAATAACAATGCCTTGAATAAAGCATGAGTTAATAAATGAAAAAAAGCCAAATCTGAAAAACCTAATGATAAAATTCTTATTATTAATCCTAATTGACTTAAAGTTGATAAAGCAACAATCTTTTTTAAATCATATTCAAAATTTGCTCCTAACCCTGATATAAACATTGTTAAAACAGATATTACTAGTAAAAACTTTAATAATCAATCCGGAAAAACCTTACAAAATCGGATTAATAAATAAACCCCAGCCGCAACCAAAGTTGATGAATGAACTAGGGCTGAGACAGGAGTTGGAGCAGCTATTGCTGCAGGCAATCAAGCTGAAAAGGGAATTTGTGCACTTTTTGTTATTCCTGCTAAAAGTACTAAAAATGAAATTAAATATATTTCAATTTCATTTAATGTACAATCTAAATAAAAAATATAATTTCATCTTCCAAAATTTAATATTCATGAAATTGCTATTAATAAAGCAACGTCCCCTACTCGATTAGATAAAGCTGTCAATATTCCAGCATTATAAGACTTAGTATTTTGATAATAAATTACAAGACAATAAGAAATTAAACCTAACCCATCTCAACCTAATAAAATTCTAATCAAATTTGGACTAATAATTAAAAACATTATAGACAATACAAATATTAATACTAAAAAAATAAACCGATTTAGTGATGAATCTCCAGTTATATAATCTTCTCTATATAAAATTACTAAGGATGAAATTAATAAAACAAAACTCATAAATATGAGAGACATTCAATCCAACAATATTGTTATAACAACTGAAGAAGTACTTAATCTAACAATTTCTCATTCAATAAAAATAATTAAATCATTTAAAATAAAAACCATTCTAGAAATAAATATTATTAAAGAAAATAATGCTAATAAAAAAAATCTAATAAAACATAATGATAAATAAACCATAACTTAAAATAACTATTCATTATATCCATGATACCACAAATCATAATTTTATGATAAACTATTTAAGTAAATTAACTTTTATTTAAAATCAAATTAAAACATAATCTCTCTTTAAAATTAATAAATTTAAGGGTAATCAATGAAGTATTATTAATAAATATTCACGTCTATACCCTCCAACTCTTCTATAAATGCCTGAATAATAAAATCCATGTTGACTATATGAATATAAATATAAAGTATAAGCAGCTCTAAAAAATGAAATTAATATAAGAAAAAATATAGATATTCATACCCAACTTACTATACTATTGAATAATCCAATTTCTCCTAATAAATTTAAAGTAGGGGGAGCAGCTATATTGCTAGATGAAAGTAAAAATCATCATAATGTTAAACTTGGCATCAAATTTAATAAACCCTTATTAATTAACAATCTTCGTCTTCCCAGACGCTCATAACTAATATTAGCTAAACAAAATAAACCAGATGAACATAAACCATGAGCAATTATTATTACAAAAGTTATATAAAACCCTCAAATATTTAATGTTATTAAGCCCCCAATAATTAAACCTATATGAACCACAGACGAATAGGCAATTAATGCTTTTACGTCTATTTGACGTAAACACATTAAACTAACTATAACCCCACCGATTAATCTAGTAGAAAATCAAAATACATTAACTAATATCCCAACTCTCTTCAAGAATTCATATACTCGTAATAAACCATAACCTCCTAACTTTAATAAAACTCCAGCTAAAATCATTGAACCTGAAACTGGAGCTTCAACATGAGCCTTAGGTAATCATAAATGCACTAAAAATATAGGCATCTTAATCAAAAAAGCTAAAATTATTCTAAAATATAAATATCAATTCAATAAATTATTTTTATAAACTAACGAAAAAACTAAATAGCCCATGTTGTTATAAAAATATATAATACCCATCAATAATGGTAATGAAGCTAAAAGAGTGTATAATAATAAATAAATACCAGCTTGCAATCGCTCAGGTTGATATCCTCACCCAAAAATTAAAAATAAAGTTGGAATCAAACTACCCTCAAAAAAAAAATAAAATGAAATGAAATTTATTCTACAAAAAGTACAAATTAATATCAATAACAATATTAAAATTATAAATATAAATAAATTATTATAAAATTTATATCGAATAACAGAATAACTAGCTAAGATTATTAAAACACAAATTCAAAATCTTAGCACAATAAGACCATATGATAAGTAATCATATCCAAATATATAACTCATTCTTATTCAATAAAAAAAATCCACTCCAATAAAAAATTTAAAAGATATAAAAAATAATAAATTCTGAATTAATAATCAATTTTTATTCATTAAACATAATGGAATCAAAAACATTAAACTTAAAATATATCTTAACATTGCAATAAGCTAAACGAATTAAAATAATCATTTCCATGAGTTCGAATTATAGAAACCAAAATTGATAATCCTAATACACCTTCACAAACTGCAAATGAAAGAAAAATTATAGTCATATATAACTCTCCTCTTATTATTAAATAAAAATACAAAATAATGAACAAAACTAAAACAATAAATTCTAAACTTAATAAAGTTACTAATAAATGTTTACGTCCAGAAGAAAAAACTCACAAACCACATAAAAATATAAAACAGAATATTATTAACATTTTTTAGTTTTAATAGTTTACTAAAAACACTGGTCTTGTAAACCAAAATTAAGAATAATTACTTTTAAAACTTCAAAGGAAAGAAACTCATCATTAATTCCCAAAATTAATATTTTATTATTAAACTACCCTTTGATATTTTATACTCATTATTAACTATAAGTTTAATTTTAAGAATTACTTTGATATTTTTAAATCATCCAATATCTATAGGTTTTATCCTTTTTATTCAAACGTTATGCTTATGTTTTATTAGAGGTTTTATATCACTAAGATTTTGATTCTCCTATGTATTATTATTAATTTATTTAGGAGGAATATTAATTTTATTTATATACATTACCAGATTAGCCTCAAACGAATTATTTTCTTATTCAAATAAAATCTTATTAATTATTTTTTTAGTACCCCTTATCTTAAGACTTATCCATTATACAAGTTTTTATCATCAAACAAACTTATATGAAAATATAGAAAATAGAATAAATTTCATCTTTATACCAAACAACTTTTTATTAAAGATATATAATTATCCTAATAACATTATTACAATCTTAATTGCCTGTTATTTATTTTTAGCTTTAATTGCAGTTGTTAAGATAACAAATATTTTCAAAGGACCCCTACGACAAATAAATTAATAATGTTTAAACCCTTACGAAAAACTCACCCTATCATCAAAATTTCCAATAATGCCTTAGCAGATTTACCTTCACCATCAAATATTAGATCATGATGAAATTTTGGTTCCCTTCTAGGCTTATGTTTAATTCTTCAAGTAATTACAGGACTATTTTTAGCTATACACTATTCAGCTCATATTGATTTAGCCTTCTCAAGAGTGATTCACATTTGTCGAGATGTAAATTATGGGTGACTTTTGCGAATTCTTCATGCTAATGGTGCTTCAATATTTTTTATTTGTATTTATCTCCATATTGGACGGGGGATATATTACGGATCATATAAATTTTATTATACATGAATAATTGGAGTAATAATTCTATTTTTATTAATAGCGACAGCATTTATAGGTTATGTATTACCATGAGGTCAAATATCATTTTGAGGTGCCACAGTAATTACTAATTTATTATCTACTATTCCCGGATTTGGAAATGAATTAGTTCAATGAGTTTGAGGGGGTTTTGCAGTAGATAATGCTACCCTAAACCGATTTTTCACATTTCATTTTATTTTACCATTTATTATTGTAGCAATAATTGCAACCCACTTATTATTTCTTCACCAAACTGGATCAAATAATCCCTTAGGTACTGATAGTAATATTGATAAAATTCCGTTTCACCCTTATTTCACATTTAAAGATATCTTAGGATTCATTATATTATTAACACTTCTATCCCTCTTATCTCTTAAAGAACCCTATATCCTAGGAGATCCTGATAATTTTATTCCTGCTAACCCTCTTGTTACTCCAGTCCACATTCAACCAGAATGATACTTTTTATTTGCTTATGCTATTTTACGCTCTATCCCTAACAAGCTAGGAGGGGTGATTGCTCTAGTAATATCTATTATAATTCTAATTATTATACCTTTCATAGATACTAACTTACGAAGATTTCAGTATCATCCTATTAATCAATTCATATTTTGATTTATAATTATAACTATTACACTTTTAACATGAATTGGAGCACGCCCTGTTGAAGACCCTTTTATCCTAGTAGGACAAATTCTCACCATTATATACTTCCTTTTCTATATCATAAACATTTTAATTATCAAGGTATGAGACCATATTATACAATAATAAATAAGTTATAAACTTAATGTTAAGATTATGTCTTGAAATCATAATAAAGGGGTTAAATTCCCTTATTAACTTTACTTCATCCAACCCTTAAAATAAAATTTTTAACCCTAAATAAAAAAACAAAAAATTTAAAGATAAAGGTAAAAAGCTTTTTCACGCTAAATATATTAATTTATCATATCGATATCGAGGAAGAGTTCCACGAACTCAAATATATATAAAAGCAATAAAAATCAACTTCAAATAAAATACAAATGAATCCAAATTAGAACCCAAAAAAATTACACATATTAACATTCTTATAAATAAAATTCTTGAATATTCTCCTAAGAATATTAATGCAAAGCCTCCCCCTCCATACTCAATATTAAAACCTGAAACTAATTCTGACTCTCCTTCAGCAAAATCAAAAGGTCTACGATTTGTTTCTGCTAAACATGACACTATTCACACATTACACAATGGTAAGTATAAAAATAAAAATCAAATATTCATCTGATAATAAAAAAAATCCATTAATGAATAACTTCCAATTAAAAAAACAAAAGATAATAAAATTAAGGCTAATCTTACCTCATAAGAAATTGTCTGAGCAATTGCTCGTAAACTCCCTAATAAAGCATAATTAGAATTTGAAGATCATCCAGCTAACATAACTGCATAAACACCTAAACTTGTACATACTAAAAAAAACAACAAACCCAAATTAAAAGTAAATAAACCTCTTATATAAGGAAATAATATTCATAAGATTAAAGATAAAAATAAAGTAAATATTGGACATACATAATATAATGAATAATTCGAAACTAAAGGATTTATATATTCCCTACAAAACAACTTTACAGCATCACTGAATGGTTGTAAAATACCAATAAATCCAATCTTATTAGGACCCTTACGAATATGAATGTATCCTAAAACCCTACGTTCTAATAAAGTAAAAAAAGCTACCCCAACTATAACAAAAATAACTAAAATTAACCCTACTAATATTATTAAAAGTACTTCACTAATTAACATTTACTATCTATAATTCAATTTATCTATTTAGGTTCTAAACCTATTACACTTTACTCTGCCAAAATAGTTTTATTAATAATATTCTTTTTCCTTCATAAAAATTATTTCAAATATTTGGTCCTTTCGTACTAAAATATTCTAATTAATTAAAGATAGAAACCAACCTGGCTCACGCCGGTTTAAACTCAGATCATGTAAGAATTTTAAGGTCGAACAGACCTAGTTATTAAACATCTACATTCAAAACTAATCTTAATCCAACATCGAGGTCATAATCTTTTTTTTCAATATGAACTTTCAAAAAAAATTATGCTGTTATCCCTAAGGTAATTTAATCTTCAAATCATTAACCATGGATCTTAAAACTTAATATAATATTAAACTCAAAAAAGAGTTCATTTTATCTTTTAATCACCCCAATTAAATATATTTCACATAGATAATAAATTCATTATTTTAACTTTATTAAATTCAAATATATATTAAACTCTATAGGGTCTTCTCGTCCCTTAATTTTATTTAAGTATTTTTACTTAAAATCTAAGTTAAATTAAATTATTATAAAATAGTCTTTACTTCATCCAACCCTTCATACTAGCCCTCAATTAAAAGACTAATGATTACGCTACCTTTGCACAGTCAATATACTGCGGCCCTTTAAACCACATTCAGTGGGCAGGTTAAATCTCTTATTTCCCCAAGAAACCATGTTTTTAATAAACAGGTGAGTAAATTATTTGCCTAATTCCTCATATTAACATTACATTTCATTCACAAAATAATCCAGACTTTACTAATTAAATCATACTTACTCACTTTTTTTTATTTAACTTAACATTTTAATATACATACTAAATAATAACTAAAAATTTTAAACAAAATAAGAATTAAATTTCAACATCCTTAAATTAATAACAAATTCTAAATTTATACAATTCTTCATTAATAAATTATTATAAATATTCTTCAAAAAGATTTTCCCTTTTAAAGTCAAGAAAACAAAATATTTAAATATTTAAATATTTAATAAATTATATTCTCTAAATTAAATCTATTTATTAAAAAACTAGATATTATTGAAAACGATTAACATTTCACTACTAATTAATAATTCTTAATATTTATAATACAATAATTAAAACTATTAATTAAATCTTTCAAATTCAAGAAAAAAAAAATTAATTATTAATTTCGATATACTCTGAAACACAAGATACAATAAAAAAAATCAACTTAAATTAATTATATAATATTAAACTTCTTTTACAATACTAATTTACTATAGTATTAATTATTATCTCAATTGATAATACAATAACAAAATTTTAATTAATTTATATTCTAAATAATATCAAATCAAAAACAATCTTATGAATTTCAAATTATATTGAATTGCACAATAATAATTTTCAATGTAAATGAAAATCCTTACCTTTAAGTTTAATTTGTTCAAGTGACTTTCTAGGCACACCTTCCGGTACGCCTACTTTGTTACGACTTATCTCATCTTAATATGAGAGTGACGGGCGATGTGTACATATTATAGAGCTATTAATCATTTTAACAAACTTCATAAAATTACAATTAAATCCACCTTTAAATTACTTTCCAATAATTATCCATAATAAATACAATTTATTGTAGTCCATCATCAAACTTACTTATTACTGCACCTTGACTTGAAATATTATATATTTTTATGCTCAGAAAATTATCTAAAAAAATATTCTCAAACAGCGGTATACAAAAATATAAAGTAAGTAAGGTCCAACGCGGATTATCAATTAAATAATAGACTCCTCTAAATAGGTTATAATACCGTCAAATTCTTTAAGTTTCAAGATCATATCTGCTAATACTTTAGTTTATTAATTTACCATAAAAATAATAGGGTATCTAATCCTAGTTTACCTTTTTAATCTCATAATCCATCATACTAACTAATAATTAATTAAAATATTTTAAAAACATTTCACCTTAAAATACTTTAATCATCATTTAACATTAATTAACATTAATTATTTAAAAACCAATATTATTTATTTAATTTCACTGTATCACCGCGGATGCTGGCACAATTTTTACCAAACTTTTATAACATTCACTAAATCTAAAATAATTTAATATCAATAAAATTACCTACTGCACAATTAACTTTATAAACCTCATTGAGAAAACTATAATATACACACATTCACATTCAGAATAACATTAAAACAAATAATCAAAGCAAGAATAAAACTAAATATTTTTTCCAAAAATCGAAGACCTGACATTGGAACACCTAACTTTTTTAATGATTTTTGTATACCTACAAATATTTGATTAAATTAATCATTGCTTCAATATATAAACTTTCAAAAAAAAAATCCACTTTACCCAAAAAATAAGTATGCTTTTTTTAAAACGTTTTCCTCCCTAGAAGTAGGAATTAAGTGCCCAATTCAATTCGATTTAATGTTTTTTTTTAAACATTGGTCTACTAAAGTATATTTAGTATATAATAAATTTAATTATATATATGTCTTATCAAAGATTAATAAAAAAAATATTCTCAAACAGCGGTATACAAAAATATAAAGTAAGTAAGGTCCAACGCGGATTATCAATTAAATAATAGACTCCTCTAAATA

4. I recommend one more round of editing for writing clarity. For example, Lines 50-51 are a little confusing, and should probably be split up into two sentences.

Reply: Thanks for your suggestion. Lines 50-51 have been split up into two sentences. Please check lines 56-57.
